# A Multidimensional Profile of Quality of Life in Patients Living with Long-Term Mechanical Circulatory Support

**DOI:** 10.3390/jcm15176907

**Published:** 2026-09-07

**Authors:** Zuzanna Strząska-Kliś, Małgorzata Sobieszczańska-Małek, Łukasz Czyżewski, Maciej Kuśmierczyk, Mariusz Kuśmierczyk

**Affiliations:** 1 Clinic of Cardiac, Thoracic and Transplant Surgery, Medical University of Warsaw, 02-097 Warsaw, Poland; malgorzata.malek@uckwum.pl (M.S.-M.); maciej.kusmierczyk@uckwum.pl (M.K.); mariusz.kusmierczyk@uckwum.pl (M.K.); 2 Geriatric Nursing Department, Medical University of Warsaw, 02-097 Warsaw, Poland; lukasz.czyzewski@wum.edu.pl

**Keywords:** mechanical circulatory support, heart failure, quality of life

## Abstract

**Background/Objectives:** Despite advances in diagnostics, prevention, and therapy, heart failure (HF) remains the leading cause of death among cardiovascular diseases. The prognosis for patients remains unfavorable, and their quality of life is severely reduced. In end-stage HF, the only remaining treatment options are heart transplantation (OHT) or the use of long-term mechanical circulatory support (MCS). **Methods:** This study had a cross-sectional, observational design. Three research tools were utilized: (1) an original metric–clinical questionnaire covering sociodemographic variables (age, sex, education, weight, height, place of residence) and self-reported clinical data (Left ventricular assist device (LVAD) pump model, date of implantation, controlled parameters, comorbidities, estimated ejection fraction, physical activity); (2) the Minnesota Living with Heart Failure Questionnaire (MLHFQ)—a validated, standardized, and translated into Polish anonymous questionnaire; and (3) the Short-Form Survey (SF-36)—a validated, standardized, and translated into Polish anonymous questionnaire. **Results:** The conducted analysis revealed that patients treated with MCS demonstrated higher quality of life (QoL) across all analyzed aspects. The effect size for the differences was moderate (for bodily pain assessment) or strong (for all other dimensions). The presence of LVAD support explained the largest percentage of variance in QoL, determining the QoL score at 21.9%. **Conclusions:** The use of MCS is associated with a significantly higher patient QoL in all analyzed dimensions compared to individuals without support. The obtained results emphasize the importance and role of LVAD therapy as an effective therapeutic option associated with higher QoL in patients diagnosed with end-stage HF, which serves as a compelling argument in clinical decision-making regarding qualification for treatment.

## 1. Introduction

Heart failure represents a leading epidemiological and clinical challenge of the 21st century. In Poland, it affects approximately 1.2 million people. HF is characterized by high mortality, the necessity for frequent and recurrent hospitalizations, and a complex therapeutic process. The progressive nature of HF is associated with a gradual decline in functional capacity and a significant reduction in patient quality of life [1,2]. Due to the aging population and the chronic course of the disease, the number of patients with HF is steadily increasing, making it a major public health issue. Delayed diagnosis or a lack of comprehensive management is associated with a poor prognosis. Citing the European ESC-HF Pilot Study in a population of hospitalized HF patients, the 12-month all-cause mortality rate was 17%, while the rehospitalization rate during this period reached nearly 44% [3].

Comprehensive assessment of heart failure severity in clinical practice relies on the complementary application of two fundamental classifications: the American College of Cardiology (ACC)/American Heart Association (AHA) staging system and the New York Heart Association (NYHA) functional class. The ACC/AHA classification (stages A–D) focuses on objective, structural disease progression, depicting its unidirectional continuum—from the presence of risk factors alone, through asymptomatic dysfunction, to refractory end-stage heart failure [4]. Conversely, the NYHA scale (classes I–IV) is dynamic in nature and is used to evaluate the patient’s current functional status and the subjective severity of symptoms in response to physical exertion. Advanced heart failure with reduced ejection fraction (HFrEF) in stage D according to the ACC/AHA classification and class IV according to the NYHA is associated with substantial morbidity, reduced quality of life, diminished functional capacity, and high mortality. The one-year survival rate for patients with advanced HF treated solely with guideline-directed medical therapy (GDMT) is merely 6–25% [5]. The primary goals of managing advanced HF are reducing mortality, alleviating disease symptoms, improving QoL, decreasing the frequency and necessity of hospitalizations, and preventing disease progression. Both etiology-targeted and symptomatic treatments are essential. For patients presenting with HFrEF, the “gold standard” involves neurohormonal antagonists (ARNI, beta-blockers, and mineralocorticoid receptor antagonists), complemented by diuretics for symptomatic relief [6]. Supplementary treatments include SGLT2 inhibitors, ivabradine, and digoxin, whereas in decompensated HF, inotropic agents or nitrates are utilized. The 2021 European Society of Cardiology (ESC) guidelines for the diagnosis and treatment of acute and chronic HF emphasize the necessity of implementing non-pharmacological interventions in patient care. These encompass multidisciplinary, interdisciplinary team-based care, patient education, engagement in self-monitoring of health status (including adjustments to diuretic therapy), post-discharge follow-up care (regular visits, access to telephone support, and remote monitoring), and enhanced accessibility to primary healthcare services [7]. In contemporary comprehensive HF care, the role of telemedicine and remote monitoring is increasingly recognized as being as critical as optimal pharmacotherapy, facilitating continuous patient evaluation and connecting care pathways [8,9]. In the management of advanced HF, interventional and surgical procedures play a crucial role in improving hemodynamics and patient prognosis. Corrective cardiac surgery provides etiologic treatment for patients whose heart failure stems from reversible structural or ischemic changes. These procedures primarily include coronary artery bypass grafting (CABG) in patients with advanced coronary artery disease, as well as valve repair or replacement (e.g., in cases of severe mitral regurgitation or aortic stenosis). Furthermore, the implantable cardioverter–defibrillator (ICD) serves as the cornerstone for the prevention of sudden cardiac death (SCD), to which patients with HFrEF are particularly predisposed [10].

When standard pharmacological treatment, inotropic agents, optimization of left ventricular inflow and outflow, and cardiac resynchronization therapy prove insufficient, some patients require temporary or long-term MCS. LVAD currently represents a viable therapeutic alternative for a select group of patients, offering the potential to extend survival and enhance quality of life. The latest clinical trials demonstrate two-year survival rates exceeding 80%, which is comparable to early outcomes following heart transplantation, thereby establishing LVAD implantation as a meaningful alternative for appropriately selected patients [11].

A candidate for LVAD implantation should be evaluated in terms of NYHA functional class and the severity of heart failure according to the Interagency Registry for Mechanically Assisted Circulatory Support (INTERMACS) scale. One-year survival rates for patients receiving the device during cardiogenic shock (INTERMACS 1) are the lowest at approximately 52%, compared to 63% in INTERMACS 2, 78% in INTERMACS 3 or 4, and 93% in INTERMACS 5. Therefore, early qualification at the outpatient level is crucial, while the patient’s general condition allows for surgery, thereby avoiding or minimizing perioperative complications associated with systemic critical illness [12]. Currently, the HeartMate 3 system is the only FDA-approved device for long-term left ventricular support in adults [13]. It is a continuous-flow pump featuring a fully magnetically levitated rotor designed to minimize thromboembolic complications. This support system can serve as a bridge to transplantation, a bridge to recovery, or destination therapy.

The application of this technology demands greater patient understanding and active engagement in their therapeutic process. Beyond standard medical adherence, patients must acquire the skills to operate the implanted system and adapt to living with an external controller and associated lifestyle restrictions. The involvement of a Ventricular Assist Device (VAD) Coordinator is particularly beneficial in this context [14]. These are trained healthcare professionals from specialized centers who maintain systematic contact with patients, offering rapid responses to reported difficulties and instilling confidence in daily life with the device [15]. Education provided by the Coordinator enhances the sense of security, enables better coping with therapy-related limitations, and promotes patient autonomy in daily living. By offering psychosocial support, the VAD Coordinator plays a pivotal role in the interdisciplinary care model for mechanical circulatory support, serving as a vital link between clinical supervision and continuous outpatient care. They are responsible for structured patient and caregiver education regarding MCS management, including driveline exit-site care, alarm interpretation, and emergency protocols. Through continuous hemodynamic and technical monitoring, alongside prompt intervention upon early deviations in pump parameters, the VAD Coordinator significantly mitigates the risk of complications (e.g., driveline infections, bleeding, or stroke) and reduces unplanned hospital readmission rates [16,17].

Beyond survival rates, health-related quality of life (HRQoL) assessment represents a pivotal component in evaluating the efficacy of such advanced treatment modalities. It encompasses the patient’s subjective appraisal of their physical, psychological, and social functioning. For the majority of patients, MCS implantation constitutes a major life event that profoundly impacts their daily functional status [18]. Many individuals who were previously severely restricted—primarily due to low physical capacity—find that they can resume a more active lifestyle, frequently returning to work and daily activities. However, long-term MCS support entails substantial physical, psychological, and social implications. Consequently, a comprehensive evaluation of LVAD therapy effectiveness extends beyond traditional clinical endpoints, such as survival rates or hospitalization frequency.

QoL serves as a key parameter for assessing the success of this advanced therapy, reflecting the patient’s subjective perception of how the disease and its treatment influence daily living. In the context of LVAD-supported patients, QoL evaluation assumes particular significance, as it allows for a holistic understanding of both the benefits and challenges associated with this therapeutic strategy. To objectify these measurements, standardized instruments are utilized, including the 36-item Short-Form Survey (SF-36) for generic health assessment and the MLHFQ, developed specifically for heart failure populations [19]. Routine, comprehensive quality of life assessment in HF patients should constitute an integral element of both clinical practice and scientific research. This approach enables not only a deeper insight into the disease trajectory but also the personalization of care and the integration of non-pharmacological interventions, such as psychological support, cardiac rehabilitation, and palliative care.

## 2. Materials and Methods

The study included 264 adult patients (>18 years of age) with advanced heart failure (NYHA functional class III/IV), divided into two cohorts. Importantly, all patients in both the LVAD and the control groups were classified as NYHA functional class III or IV at baseline. The first group comprised 142 patients (132 males, 10 females) undergoing LVAD support. In this group, 100% of the patients were supported with the HeartMate 3 device, and the duration of LVAD support ranged from 1 to 500 days. The second group consisted of 122 patients (87 males, 35 females) serving as a control (comparative) cohort receiving medical therapy alone (without mechanical circulatory support), pending decisions regarding surgical intervention (HTx/MCS) or continuation of pharmacological treatment. The baseline characteristics of the study group are presented in Table 1.

Data collection was conducted between January 2023 and December 2024, maintaining full anonymity of the respondents. An electronic questionnaire created via Google Forms served as the research tool. The survey was distributed through a dual-track approach: via email messages containing a link to the study and through social media platforms, where the form was shared on dedicated patient forums (including the VAD Polska support group). Non-participation in the survey was strictly due to either direct patient refusal (lack of consent) or the patient meeting one of the predefined exclusion criteria. The inclusion and exclusion criteria for the study are summarized in Table 2. The study was initiated following approval from the Bioethics Committee of the Medical University of Warsaw (Approval No. AKBE/24/2024, dated 15 April 2024).

Statistical calculations were performed using IBM SPSS Statistics software (version 29.0). This quantitative, observational, cross-sectional study evaluated HRQoL using three measurement instruments: the SF-36, the MLHFQ, and a custom-designed questionnaire. For the SF-36, we utilized the validated Polish adaptation developed by Tylka and Piotrowicz (2009) [20]. In accordance with this validation, a reverse coding strategy was applied to harmonize the directionality of the SF-36 with the MLHFQ, ensuring that for both instruments, lower scores consistently indicate less severe symptoms and better quality of life.

Initially, basic descriptive statistics were computed for quantitative variables representing QoL dimensions. The normality of data distribution was assessed using the Kolmogorov–Smirnov test, alongside the evaluation of distribution parameters, including skewness and kurtosis. Categorical variables were compared between groups using the **χ^2^** test of independence. For comparative analysis of quantitative variables between the two cohorts, the independent-samples *t*-test was applied.

To identify factors associated with QoL in the study population, univariate and multivariate linear regression analyses were conducted. Furthermore, a two-way analysis of variance 2 × 4 was performed to evaluate QoL levels based on physical activity engagement and LVAD support status. The Bonferroni test was utilized for post hoc pairwise comparisons. For all statistical tests, a significance threshold of α = 0.05 was assumed.

## 3. Results

To evaluate HRQoL, an independent-samples *t*-test was conducted to determine whether patients supported with LVAD differed in their QoL assessment compared to control group patients (NYHA class III/IV) without mechanical support (Table 3). The analysis revealed that individuals with an LVAD presented significantly higher QoL across all evaluated domains (mental component, physical component, and overall quality of life). The effect sizes for these differences ranged from moderate (for bodily pain assessment) to strong (for all other dimensions). In accordance with the Polish validation guidelines for these questionnaires, lower numerical scores indicate a higher QoL in a given domain. These results demonstrate that patients implanted with HeartMate 3 rated their physical and social functioning higher, experienced fewer role limitations due to physical health or emotional problems, and reported lower bodily pain severity. Furthermore, they exhibited a greater sense of overall physical and mental health, as well as higher vitality levels.

Univariate and multivariate linear regression analyses were conducted to determine the factors associated with QoL. Having an LVAD was identified as the variable most strongly associated with quality of life, accounting for 35.3% of the variance in the overall SF-36 index and 21.9% of the variance in the MLHFQ score. Other significant predictors of higher QoL included age (younger patients achieved better outcomes) and physical activity (more frequent exercise improved both physical and mental functioning). Conversely, the presence of comorbidities (explaining approximately 5% of the variance) had a negative impact on QoL. Detailed analysis of variance (ANOVA) confirmed that patients exercising 2–4 times per week demonstrated the highest quality of life. In the subsequent step, a series of univariate linear regression analyses using the enter method were performed to identify which demographic factors and clinical parameters were associated with QoL. The predictors included in the models were sex, age, place of residence, presence of LVAD support, etiology of heart failure, presence of comorbidities (such as arterial hypertension, type II diabetes, cardiac arrhythmias, and atherosclerosis, among others), left ventricular ejection fraction (LVEF), cigarette smoking, and physical activity engagement. The dependent variables were defined as four main indicators: QoL measured by the MLHFQ and three SF-36 dimensions—the overall QoL index, physical component summary, and mental component summary.

Table 4 presents the regression analysis results for factors associated with QoL measured by the MLHFQ. As shown in the data, age, presence of LVAD support, presence of comorbidities (including other conditions), cigarette smoking, and physical activity showed statistically significant associations with QoL assessment.

Another evaluated dependent variable was the overall QoL index (Table 5). Statistically significant predictors of overall QoL included the same set of variables as for QoL measured by the MLHFQ. Additionally, sex and heart failure etiology (other than ischemic cardiomyopathy) emerged as significant predictors of the overall QoL index. A detailed analysis of the obtained results demonstrated that male sex, younger age, and the presence of LVAD support were associated with higher QoL. Furthermore, patients diagnosed with ischemic cardiomyopathy exhibited higher QoL compared to those with other underlying etiologies. Conversely, the presence of comorbidities (including conditions other than those specifically analyzed) was associated with lower QoL. Cigarette smoking likewise reduced QoL, whereas more frequent physical activity enhanced it. These findings align with the previously reported data.

The strongest association was observed with LVAD support, which accounted for 35.3% of the variance in quality of life. Physical activity explained 8.1% of the variance, while sex and the etiology of heart failure each accounted for nearly 6%. The remaining variables individually explained less than 5% of the variance in quality of life.

**MLHFQ Quality of Life**—the first analyzed multivariate model concerned quality of life measured using the MLHFQ. The model demonstrated a good fit to the data and collectively explained over 30% of the variance in quality of life (R^2^ = 0.322; F (7) = 18.61; *p* < 0.001). As presented in Table 6, age, the presence of LVAD support, comorbidities, and physical activity emerged as statistically significant predictors of QoL. Higher QoL was associated with younger age, the presence of mechanical circulatory support, more frequent physical activity, and the absence of comorbidities.

**Overall Quality of Life Index**—the subsequent multivariate model evaluated the overall QoL index measured using the SF-36 survey. The model demonstrated a satisfactory fit to the data, accounting for nearly 40% of the variance (R^2^ = 0.387; F (9) = 18.89; *p* < 0.001). Age, the presence of LVAD support, and physical activity emerged as statistically significant predictors of the overall quality of life index. Younger age, mechanical circulatory support, and a higher frequency of physical activity were associated with higher quality of life. The results are summarized in Table 7.

## 4. Discussion

Care for patients with advanced heart failure following long-term mechanical circulatory support implantation represents one of the most demanding challenges in contemporary medicine. The complexity of clinical issues in this cohort—arising from both end-stage cardiovascular impairment and the technical challenges of managing the implanted device—requires a departure from traditional, unidirectional treatment algorithms. Optimal therapeutic management necessitates a comprehensive, multidisciplinary, team-based approach that integrates advanced surgical and cardiological supervision with continuous nursing care, psychological support, and specialized rehabilitation. Only such an organized, holistic care model ensures not only safe patient adaptation to living with the device but also the full realization of the clinical potential of MCS therapy, including health-related quality of life improvement [21].

Mortality and hospitalization rates remain high, and many patients progress to advanced HF in the absence of alternative therapeutic options. Cardiovascular death remains the primary cause of mortality in HF. However, over time, the proportion of cardiovascular deaths has been declining, whereas non-cardiovascular mortality is rising, particularly in patients with HF with preserved ejection fraction (HFpEF). Consequently, treatment efficacy related to patient-reported outcomes has gained increasing significance. Hemodynamic alterations, physical limitations, and the psychosocial consequences of this condition have become the focus of extensive scientific investigation. Patients frequently experience debilitating symptoms such as dyspnea, fatigue, edema, and severe exercise intolerance. Studies have demonstrated that symptom severity directly correlates with reduced QoL. In a 2015 study by Uchmanowicz et al., it was shown that HF patients with lower levels of disease acceptance exhibit significantly lower QoL [22].

The present findings demonstrate that end-stage heart failure multidimensionally and profoundly impairs patient quality of life, whereas LVAD support is associated with significantly higher quality of life scores. These data are fully consistent with global evidence from major multicenter clinical trials, such as MOMENTUM 3, which proved significant reductions in physical limitations, improvements in NYHA functional class, and alleviation of congestive symptoms utilizing next-generation magnetically levitated pumps [23]. The results obtained in this study, utilizing measurement tools including the SF-36 questionnaire, align with comparable published literature. Crucially, patients with advanced HF exhibited significantly lower QoL compared to those receiving mechanical circulatory support (Cohen’s d effect size for MLHFQ = 1.06) across evaluated domains, including physical and social functioning. LVAD-supported patients experienced fewer role limitations due to physical health or emotional issues and reported lower bodily pain severity. Furthermore, they demonstrated higher perceived physical and mental health status, as well as greater vitality (*p* < 0.001) [24,25,26]. However, when interpreting these quality of life scores, it must be acknowledged that the control group comprised patients awaiting decisions on advanced therapies (HTx/MCS). This implies a clinically different and potentially more unstable population compared to outpatients on long-term LVAD support, which may partially contribute to the observed QoL differences.

It is essential to emphasize that, given the high mortality risk associated with end-stage HF, LVAD implantation is not only favorably associated with QoL but also significantly extends survival. MCS serves as a vital therapeutic option for patients with advanced HF during qualification for heart transplantation, while on the transplant waiting list, or when transplantation is contraindicated. This is supported by landmark studies, including the 2001 Randomized Evaluation of Mechanical Assistance for the Treatment of Congestive Heart Failure (REMATCH) trial, which demonstrated that advanced HF patients managed with MCS exhibited superior 1-year and 2-year survival rates compared to medical therapy alone (52% vs. 25% and 23% vs. 8%, respectively), alongside improved QoL [24]. The mean age of evaluated patients was 51.96 years in the medical therapy group and 55.94 years in the support group, confirming extended survival post-LVAD implantation. According to 2017 INTERMACS registry data, approximately 20% of MCS-supported patients remained free from major adverse events—such as infection, bleeding, stroke, device malfunction, or death—at 3 years. Overall Kaplan–Meier survival during support reached 81% at 1 year and 70% at 2 years. Substantial improvements in quality of life persist for at least 2 years following HeartMate 3 implantation, with approximately 80% of patients reporting positive overall experiences with LVAD therapy during the first 2 years. The occurrence of major adverse events within the initial 3 post-implant months significantly influences long-term survival and therapeutic efficacy. Patients with severe HF (NYHA class IV) managed without interventional therapy (including LVAD) face 1-year survival rates of 30–50%, declining to 10–30% at 2 years. Long-term observational data indicate that 5-year survival for advanced HF patients receiving neither MCS nor heart transplantation is merely 5–10% [26].

However, it must be noted that QoL improvement in the mental domain was slightly less pronounced than gains in physical functioning. This disparity may stem from the specific psychological profile of LVAD patients, in whom physical recovery is frequently accompanied by anxiety disorders, depression, or a perceived loss of autonomy. Living daily with a mechanical device, fear of technical failure, driveline care requirements, infection risks, and awareness of thromboembolic or hemorrhagic complications impose a substantial psychosocial burden. Patients often experience persistent apprehension regarding the reliable performance of the life-sustaining apparatus.

These challenges underscore the paramount necessity for multidisciplinary, multifaceted care in LVAD recipients. The VAD Coordinator plays a central role in treatment orchestration, patient and family education, and early complication detection via remote telemonitoring systems. Generic assessment instruments, such as the SF-36, do not fully capture the unique psychological nuances of device-dependent patients, highlighting the growing clinical need for highly specialized evaluation tools. Additionally, a positive correlation was observed between physical activity levels and higher QoL scores, supporting the systematic implementation of tailored cardiac rehabilitation programs following pump implantation [27,28].

Regarding physical activity, while active patients reported better QoL, this relationship should be interpreted with caution. Reverse causality is highly plausible; patients with a better baseline QoL and clinical stability are inherently more capable of engaging in physical activity. Furthermore, our recruitment method—utilizing the internet group of the only national patient association for mechanical circulatory support in Poland—may introduce digital literacy and survivorship biases. However, it should be emphasized that a certain degree of digital literacy is a practical prerequisite for LVAD qualification in Poland, as patients must independently manage the HeartMate 3 technology and communicate with coordinators.

## 5. Conclusions

1. Implantation of the HeartMate 3 left ventricular assist device (LVAD) in patients with advanced heart failure is associated with a statistically significant, multidimensional higher health-related quality of life compared to patients receiving conservative medical therapy alone.
2. The receipt of mechanical circulatory support represents the factor most strongly associated with physical functioning, whereas regular physical activity positively correlates with higher scores across mental well-being and social functionality domains.
3. Although mental component scores for quality of life were superior to those of the control group, they underscore the vital necessity of providing LVAD-supported patients with specialized psychological care to address anxiety regarding potential device malfunction and associated complications.
4. There is a clear imperative to develop and validate a disease-specific quality of life questionnaire tailored exclusively to the mechanical circulatory support cohort, capable of capturing the unique clinical and daily nuances of living with an LVAD.
5. Given the rising volume of patients undergoing device implantation, structured support within a coordinated care model (involving dedicated VAD Coordinators and telemonitoring) represents an essential element of therapy, optimizing both clinical safety and long-term patient quality of life.

**Limitations:** Several limitations of this study should be noted. First, the cross-sectional design precludes causal inferences. Second, given the specific real-world context of LVAD therapy in Poland during the study period—with only 7 certified implanting centers and a total of 285 registered LVAD patients nationwide—performing extensive adjusted analyses like propensity score matching was not feasible due to the risk of severe selection bias and loss of statistical power. Therefore, baseline imbalances between the groups remain, and our multivariable regression models should be considered exploratory. Third, Destination Therapy (DT) was not officially registered in Poland until January 2023, precluding precise stratification by implantation strategy. Finally, to guarantee absolute patient anonymity and encourage participation, clinical variables were self-reported. This precluded cross-referencing with electronic medical records for objective verification of parameters such as the INTERMACS profile or detailed pharmacological history.

## Figures and Tables

**Table 1:** Baseline characteristics of the study population.

	Cohort Group (*n* = 122)	LVAD Group (*n* = 142)	*p*
Sex, *n* (%)			
Female	35 (28.7%)	10 (7.0%)	<0.001
Male	87 (71.3%)	132 (92.3%)	
Age, *M (SD)* Min–Max	51.96 (13.77) 18–80	55.94 (10.97) 18–73	0.011
Weight, *M (SD)* Min–Max	81.39 (15.39) 49–125	87.42 (12.70) 55–12,080,117	<0.001
Height, *M (SD)* Min–Max	177.17 (13.39) 157–281	179.37 (9.10) 160–204	
Place of residence, *n* (%)			
Village	30 (24.6%)	25 (17.5%)	0.340
City with a population of up to 200,000	46 (37.7%)	56 (39.2%)	
City of population over 200,000	46 (37.7%)	62 (43.4%)	
Duration of mechanical circulatory support		32.35 (50.94) 1–500	
Cause of heart failure, *n* (%)			
Ischemic cardiomyopathy	36 (29.5%)	80 (55.9%)	<0.001
Dilated cardiomyopathy	29 (23.8%)	41 (28.7%)	
Other	57 (46.7%)	22 (15.4%)	
Concomitant diseases, *n* (%)	105 (86.1%)	114 (79.7%)	0.321
Hypertension	17 (13.9%)	26 (18.2%)	0.405
Diabetes II	29 (23.8%)	38 (26.6%)	0.671
Mitral regurgitation	3 (2.5%)	2 (1.4%)	0.664
Aortic regurgitation	1 (0.8%)	0	0.460
Bronchial asthma	6 (4.9%)	9 (6.3%)	0.791
Kidney failure	12 (9.8%)	3 (2.1%)	0.008
Hypothyroidism	9 (7.4%)	6 (4.2%)	0.296
Cardiac arrhythmias	28 (23.0%)	31 (21.7%)	0.882
Atherosclerosis	23 (18.9%)	21 (14.7%)	0.409
Gout	7 (5.7%)	13 (9.1%)	0.356
Other	64 (52.5%)	57 (39.9%)	0.048
Depression	1 (0.8%)	2 (1.4%)	1.000
Pulmonary hypertension	6 (4.9%)	9 (6.3%)	0.791
Telemonitoring care, *n* (%)	3 (2.5%)	111 (77.6%)	<0.001
Cigarette smoking, *n* (%)	36 (29.5%)	13 (9.1%)	<0.001
Physical activity frequency, *n* (%)			
None	87 (71.3%)	62 (43.4%)	<0.001
1–2 times per week	25 (20.5%)	39 (27.3%)	
2–4 times per week	6 (4.9%)	17 (11.9%)	
Daily	4 (3.3%)	23 (16.1%)	

**Table 2:** Inclusion and exclusion criteria for the study.

Inclusion Criteria	Exclusion Criteria
Patients diagnosed with advanced heart failure (NYHA class III/IV) Patients post-LVAD implantation Informed consent to participate in the study Age over 18 years	Lack of patient consent Patients unable to communicate to assess quality of life

**Table 3:** Comparison of quality of life assessment between LVAD-supported patients and non-supported controls.

	Cohort Group (*n* = 122)		LVAD Group (*n* = 142)					95% *CI*		
Dependent Variable	*M*	*SD*	*M*	*SD*	*t*	*df*	*p*	*LL*	*UL*	Cohen’s *d*
Quality of life	59.37	25.94	34.46	21.21	8.46	233.61	<0.001	19.11	30.71	1.06
Physical functioning	32.90	12.25	19.15	11.89	9.24	262	<0.001	10.82	16.69	1.14
Role limitations due to physical health	14.21	6.68	6.43	6.82	9.29	259	<0.001	6.14	9.44	1.15
Bodily pain	4.71	2.13	3.04	2.17	6.28	262	<0.001	1.15	2.19	0.78
General health	14.88	3.83	10.92	3.68	8.57	262	<0.001	3.06	4.88	1.06
vitality	12.87	3.13	8.42	3.50	10.78	261	<0.001	3.63	5.26	1.33
Social functioning	4.24	1.81	2.71	1.88	6.68	262	<0.001	1.08	1.98	0.82
Role limitations due to emotional problems	9.67	5.58	3.88	5.39	8.56	262	<0.001	4.46	7.12	1.06
Mental health	13.54	4.39	8.94	3.94	8.88	243.60	<0.001	3.57	5.61	1.11
Physical component summary	75.54	22.32	45.30	20.73	11.34	259	<0.001	24.99	35.49	1.41
Mental component summary	31.49	9.59	17.96	9.99	11.15	261	<0.001	11.14	15.92	1.38
Overall Quality of Life Index	107.02	30.51	62.99	29.28	11.86	258	<0.001	36.72	51.34	1.47

**Table 4:** Summary of simple linear regression analyses for models explaining MLHFQ quality of life scores.

Variables	*b*	*SE*	β	*t*	*R* ^2^	*F*
Sex	4.13	3.35	0.06	0.95	0.003	0.90
Age	−0.31	0.13	−0.15	−2.40 *	0.022	5.76 *
Place of residence	−1.31	2.15	−0.04	−0.61	0.001	0.37
LVAD	−24.91	2.90	−0.47	−8.60 ***	0.219	73.92 ***
Cause of heart failure ^a^					0.009	1.17
Dilated cardiomyopathy	−2.59	4.02	−0.04	−0.64		
Other	3.96	3.87	0.07	1.02		
Concomitant diseases	16.05	4.27	0.23	3.76 ***	0.051	14.13 ***
Hypertension	3.69	4.43	0.05	0.83	0.003	0.70
Diabetes II	6.77	3.74	0.11	1.81	0.012	3.28
Cardiac arrhythmias	4.04	3.92	0.06	1.03	0.004	1.06
Atherosclerosis	3.82	4.39	0.05	0.87	0.003	0.76
Other	10.58	3.22	0.20	3.29 **	0.040	10.83 **
Ejection fraction	−0.003	0.01	−0.04	−0.69	0.002	0.48
Cigarette smoking	8.79	4.17	0.13	2.11 *	0.017	4.45 *
Physical activity	−10.28	1.52	−0.39	−6.77 ***	0.149	45.87 ***

^a^ Reference category: ischemic cardiomyopathy. * *p* < 0.05; ** *p* < 0.01; *** *p* < 0.001.

**Table 5:** Summary of univariate linear regression analyses for predictors of overall SF-36 quality of life index.

Variables	*b*	*SE*	β	*t*	*R* ^2^	*F*
Sex	23.75	5.97	0.24	3.98 ***	0.058	15.81 ***
Age	−0.47	0.18	−0.16	−2.57 *	0.025	6.61 *
Place of residence	−1.59	3.05	−0.03	−0.52	0.001	0.27
LVAD	−44.03	3.71	−0.59	−11.86 ***	0.353	140.66 ***
Cause of heart failure					0.059	7.90 ***
Dilated cardiomyopathy	4.37	5.50	0.05	0.79		
Other	20.85	5.32	0.26	3.92 ***		
Concomitant diseases	17.48	6.12	0.18	2.86 **	0.031	8.16 **
Hypertension	−2.00	6.20	−0.02	−0.32	<0.001	0.10
Diabetes II	1.34	5.29	0.02	0.25	<0.001	0.06
Cardiac arrhythmias	2.88	5.53	0.03	0.52	0.001	0.27
Atherosclerosis	7.68	6.12	0.08	1.25	0.002	1.57
Other	14.44	4.53	0.19	3.18 **	0.038	10.144 **
Ejection fraction	−0.002	0.01	−0.02	−0.36	0.001	0.13
Cigarette smoking	12.79	5.85	0.14	2.19 *	0.018	4.78 *
Physical activity	−10.75	2.26	−0.29	−4.75 ***	0.081	22.59 ***

Reference category: ischemic cardiomyopathy. * *p* < 0.05; ** *p* < 0.01; *** *p* < 0.001.

**Table 6:** Multivariate linear regression model coefficients for MLHFQ quality of life.

Variables	*b*	*SE*	β	*t*	*p*	95% CI for b
Sex	−5.31	3.84	−0.08	−1.38	0.168	−12.88; 2.25
Age	−0.39	0.11	−0.18	−3.36	<0.001	−0.61; −0.16
LVAD	−20.16	3.14	−0.38	−6.43	<0.001	−26.34; −13.99
Concomitant diseases	9.70	4.21	0.14	2.30	0.022	1.41; 17.99
Other	4.18	3.00	0.08	1.39	0.165	−1.72; 10.08
Smoking cigarettes	−2.51	3.62	−0.04	−0.69	0.489	−9.64; 4.62
Physical activity	−6.60	1.55	−0.25	−4.24	<0.001	−9.65; −3.54

**Table 7:** Multivariate linear regression model coefficients for the overall SF-36 quality of life index.

Variables	*b*	*SE*	β	*t*	*p*	*95% CI for b*
Sex	8.11	5.24	0.08	1.55	0.123	−2.21; 18.42
Age	−0.32	0.16	−0.11	−1.99	0.048	−0.64; −0.003
LVAD	−37.39	4.51	−0.50	−8.28	<0.001	−46.28; −28.50
Cause of heart failure						
Dilated cardiomyopathy	−1.10	4.85	−0.01	−0.23	0.821	−10.65; 8.45
Other	2.75	5.10	0.03	0.54	0.590	−7.29; 12.79
Concomitant diseases	9.86	5.75	0.10	1.72	0.088	−1.46; 21.19
Other	5.50	4.10	0.07	1.34	0.181	−2.58; 13.58
Cigarette smoking	−2.89	4.87	−0.03	−0.59	0.553	−12.48; 6.69
Physical activity	−4.51	2.18	−0.12	−2.07	0.040	−8.81; −0.21

## Data Availability

The original contributions presented in this study are included in the article. Further inquiries can be directed to the corresponding authors.

## Abbreviations

ACC	American College of Cardiology
AHA	American Heart Association
CABG	coronary artery bypass grafting
ESC	European Society of Cardiology
GDMT	guideline-directed medical therapy
HF	heart failure
HFpEF	heart failure with preserved ejection fraction
HFrEF	heart failure with reduced ejection fraction
HRQoL	health-related quality of life
ICD	implantable cardioverter-defibrillator
INTERMACS	Interagency Registry for Mechanically Assisted Circulatory Support
LVAD	left ventricular assist device
LVEF	left ventricular ejection fraction
MCS	mechanical circulatory support
MLHFQ	Minnesota Living with Heart Failure Questionnaire
NYHA	New York Heart Association
OHT	orthotopic heart transplantation
QoL	quality of life
REMATCH	Randomized Evaluation of Mechanical Assistance for the Treatment of Congestive Heart Failure
SCD	sudden cardiac death
SF-36	Short-Form Survey
VAD	Ventricular Assist Device
